# Composition and function of AChR chimeric autoantibody receptor T cells for antigen-specific B cell depletion in myasthenia gravis

**DOI:** 10.1126/sciadv.adt0795

**Published:** 2025-02-28

**Authors:** Sangwook Oh, Fatemeh Khani-Habibabadi, Kevin C. O’Connor, Aimee S. Payne

**Affiliations:** ^1^ Department of Dermatology, University of Pennsylvania, Philadelphia, PA, USA.; ^2^ Departments of Neurology and Immunobiology, Yale School of Medicine, New Haven, CT, USA.

## Abstract

In acetylcholine receptor (AChR)–seropositive myasthenia gravis (MG), anti-AChR autoantibodies impair neuromuscular transmission and cause severe muscle weakness. MG therapies broadly suppress immune function, risking infections. We designed a chimeric autoantibody receptor (CAAR) expressing the 210–amino acid extracellular domain of the AChR α subunit (A210) linked to CD137-CD3ζ cytoplasmic domains to direct T cell cytotoxicity against anti-AChRα B cells. A210-CAART incorporating a CD8α transmembrane domain (TMD8α) showed functional but unstable surface expression, partially restored by inhibiting lysosomal degradation. A210-CAART with a CD28 TMD showed sustained surface expression, independent of TMD dimerization motifs. In a mouse xenograft model, A210.TMD8α-CAART demonstrated early control of anti-AChR B cell outgrowth but subsequent rebound and loss of surface CAAR expression, whereas A210.TMD28-CAART induced sustained surface CAAR expression and target cell elimination. This study demonstrates the importance of the CD28 TMD for CAAR stability and in vivo function, laying the groundwork for future development of precision cellular immunotherapy for AChR-MG.

## Teaser

A precision cellular immunotherapy shows potential to deplete only autoimmune B cells in AChR-seropositive myasthenia gravis.

## INTRODUCTION

Chimeric antigen receptors (CARs) are a potent means of redirecting T cell cytotoxicity against antigen-specific target cells, such as CD19-expressing B cells for the treatment of B cell–mediated cancers. After target cell encounter and cytolysis, CAR T cells expand and differentiate into memory CAR T cells that can persist indefinitely to guard against cancer recurrence, leading to durable remissions and some presumed cures of B cell malignancies (*1*). Recent case reports of the therapeutic success of anti-CD19 CAR T cells in autoimmune disorders such as systemic lupus erythematosus and myasthenia gravis (MG) (*2*–*4*), among others, have generated interest in expanding the applications of the technology to treat a broad range of B cell–mediated diseases.

MG is an autoimmune disease in which autoantibodies disrupt neuromuscular transmission by binding to postsynaptic membrane proteins; specifically the acetylcholine receptor (AChR), muscle-specific tyrosine kinase (MuSK), or low-density lipoprotein receptor-related protein 4, leading to potentially life-threatening muscle weakness. Although T cells induce pathogenic autoantibody production, given that autoantibodies are produced by B cells, numerous clinical trials have been initiated to deplete B cells as a therapeutic strategy for MG (*5*). B cell depletion with rituximab, an anti-CD20 monoclonal antibody (mAb), is recommended by international consensus (*6*) as an early therapeutic option for MuSK-seropositive MG (MuSK-MG) and has been shown to reduce disease relapse and improve disease activity in some patients with AChR-MG (*7*–*9*); although randomized controlled trials of rituximab in MG have shown mixed results depending on clinical endpoint and use as first-line therapy. Recently, a patient with AChR-MG previously refractory to rituximab, bortezomib, and other immunosuppressive agents was treated with anti-CD19 CAR T cells, resulting in improvement of disease activity and a 70% drop in anti-AChR autoantibody titer within 2 months of infusion, suggesting that a substantial proportion of pathogenic autoantibodies in MG are made by short-lived plasma cells that can be targeted by depleting precursor CD19-expressing memory B cells (*2*, *4*).

We recently introduced a novel genetically engineered cellular immunotherapy named chimeric autoantibody receptor T cells (CAART) for the treatment of autoantibody-mediated diseases, including desmoglein 3 (DSG3)–seropositive mucosal pemphigus vulgaris and MuSK-MG (*10*–*12*). Additional research groups have reported the preclinical development of CAAR technology for the treatment of autoantibody-mediated forms of encephalitis and membranous nephropathy and diseases associated with anti-La/SSB antibodies (*13*–*15*). CART design incorporates an antibody to serve as the CAR extracellular domain to recognize antigens such as CD19 on target cells, subsequently triggering CART activation for target cell lysis and memory CART formation. CAART design incorporates autoantigens instead of antibodies within the extracellular receptor domain, interacting with B cells that express autoantigen-specific B cell receptors (BCRs) on their surface. Such an approach is designed to avoid global B cell depletion and instead target only the autoreactive B cell populations that cause disease. Previous studies using DSG3-CAART or MuSK-CAART have demonstrated the preclinical feasibility and safety of antigen-specific B cell depletion in in vitro and in vivo models (*10*–*12*), leading to phase 1 clinical trials in mucosal pemphigus vulgaris and MuSK-MG.

The development of CAART technology for AChR-MG poses technical challenges. Unlike DSG3 or MuSK, AChR is a pentameric complex comprising four AChR subunits, α1_2_βγδ for fetal AChR or α1_2_βδε for adult AChR. Each subunit has a multipass transmembrane structure, although the main immunogenic region (MIR) targeted by MG autoantibodies has been identified within the first extracellular domain of the α subunit (αEC1) (*16*). Here, our primary focus was to demonstrate the feasibility of developing AChR-CAART using the well-defined α subunit and validating cytolytic activity in an established experimental system.

## RESULTS

### A210.TMD8α-CAAR preserves the conformation of the MIR

**Fig. 1.**
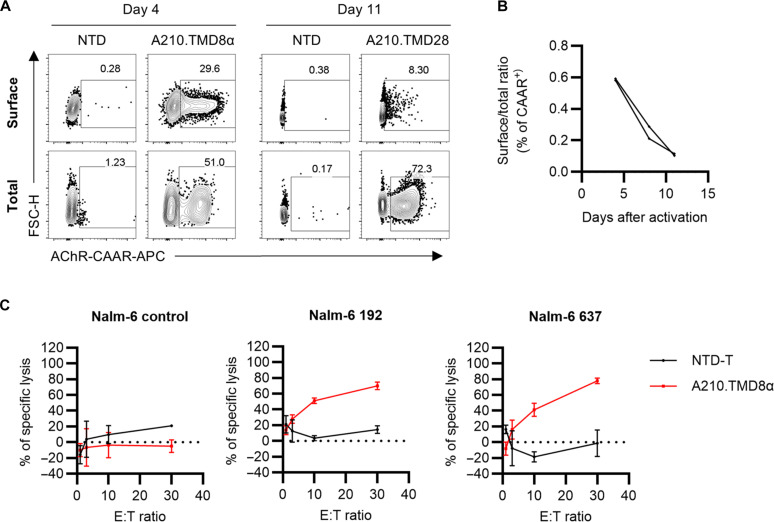
AChR-CAAR with a CD8α TMD demonstrates decreased but functional cell surface expression during ex vivo expansion. (**A** and **B**) Total and surface AChR-CAAR expression in human T cells during ex vivo expansion was detected with or without cell permeabilization, respectively. (A) Representative plots from nontransduced (NTD) or A210.TMD8α-CAAR T cells at day 4 and day 11 after T cell activation. (B) Surface AChR-CAAR expression relative to total expression is shown using two different donor CAAR T cell preparations. (**C**) A210.TMD8α- CAAR T cells were cocultured with Nalm-6 control, Nalm-6 192, or Nalm-6 637 cells for 24 hours, and specific cytolysis was measured using a luciferase-based killing assay. Each dot represents mean ± SD.

To deplete autoreactive B cells relevant to AChR-MG, the CAAR must encompass clinically relevant autoantigen epitopes. B cell epitope analysis using the Immune Epitope Database and Analysis Resource website indicates that early studies characterizing anti-AChR autoantibody specificity predominantly identified epitopes within αEC1, with the most commonly targeted epitope cluster defining the MIR. We therefore incorporated the entire EC1 domain of AChRα subunit (αEC1_1–210_ or A210) into the extracellular domain of the CAAR, linked to CD8α transmembrane domain (TMD) and cytoplasmic CD137 costimulatory and CD3ζ activation domains. The expression of A210.TMD8α-CAAR on the surface of human T cells was assessed using mAb 35 (Fig. 1A), known for binding to the MIR of the native αEC1 in a conformation-dependent manner (*17*). The detection of A210.TMD8α-CAAR in human T cells by mAb 35, as well as additional MIR and non-MIR–reactive mAbs (fig. S1), indicates that the conformational structure of the MIR within A210.TMD8α-CAAR closely resembles that of the native MIR form.

### A210.TMD8α-CAAR loses surface expression during expansion but remains functional

Unexpectedly, during ex vivo expansion, A210.TMD8α-CAAR cell surface expression decreased. Flow cytometry of nonpermeabilized and permeabilized cells indicated that cell surface CAAR expression progressively decreased, whereas total CAAR expression remained constant or increased, indicating intracellular retention of the CAAR (Fig. 1, A and B). The decline in CAAR^+^ T cells was not attributable to cell death resulting from CAAR expression, as the total percentage of CAAR^+^ T cells remained consistent during ex vivo expansion. In addition, the percentage of surface A210.TMD8α-CAAR^+^ T cells remained consistent when detected using either conformation-sensitive (mAb 35) or conformation-independent (mAb 210) anti-AChR antibodies (fig. S2) (*17*, *18*), indicating that the decrease in detection was not due to changes affecting the mAb 35 epitope.

To assess CAAR functionality in mediating cytolysis by AChR-CAART, we conducted in vitro killing assays using cocultures with Nalm-6 cells expressing BCRs recognizing the MIR of αEC1. We selected two anti-MIR antibody clones to engineer anti-AChR BCR-expressing Nalm-6 target cells, namely clone 637 (derived from a patient with AChR-MG) and clone 192 (derived from a rat) (*19*, *20*), each binding slightly different epitopes in the MIR [dissociation constant (*K*_D_) of 637: 0.0050 ± 0.0012 nM; *K*_D_ of 192: 0.0071 ± 0.0007 nM] (*17*). We first confirmed that both Nalm-6 target cell lines (637 and 192) exhibited a BCR expression level comparable to that of primary human immunoglobulin G (IgG)^+^ B cells (fig. S3, A and B). After coculturing A210.TMD8α-CAART for 24 hours with Nalm-6 cells expressing each anti-MIR antibody clone, CAART cytotoxicity was assessed using a luciferase-based killing assay (Fig. 1C). Despite the observed decline in CAAR expression on the surface of human T cells, A210.TMD8α-CAART demonstrated specific cytotoxicity against Nalm-6 cells expressing anti-MIR BCRs (clones 637 and 192), whereas no cytotoxicity was detected against BCR-negative Nalm-6 control cells, indicating functional surface CAAR expression.

### TMD modification enhances AChR-CAAR surface stability during ex vivo expansion

**Fig. 2.**
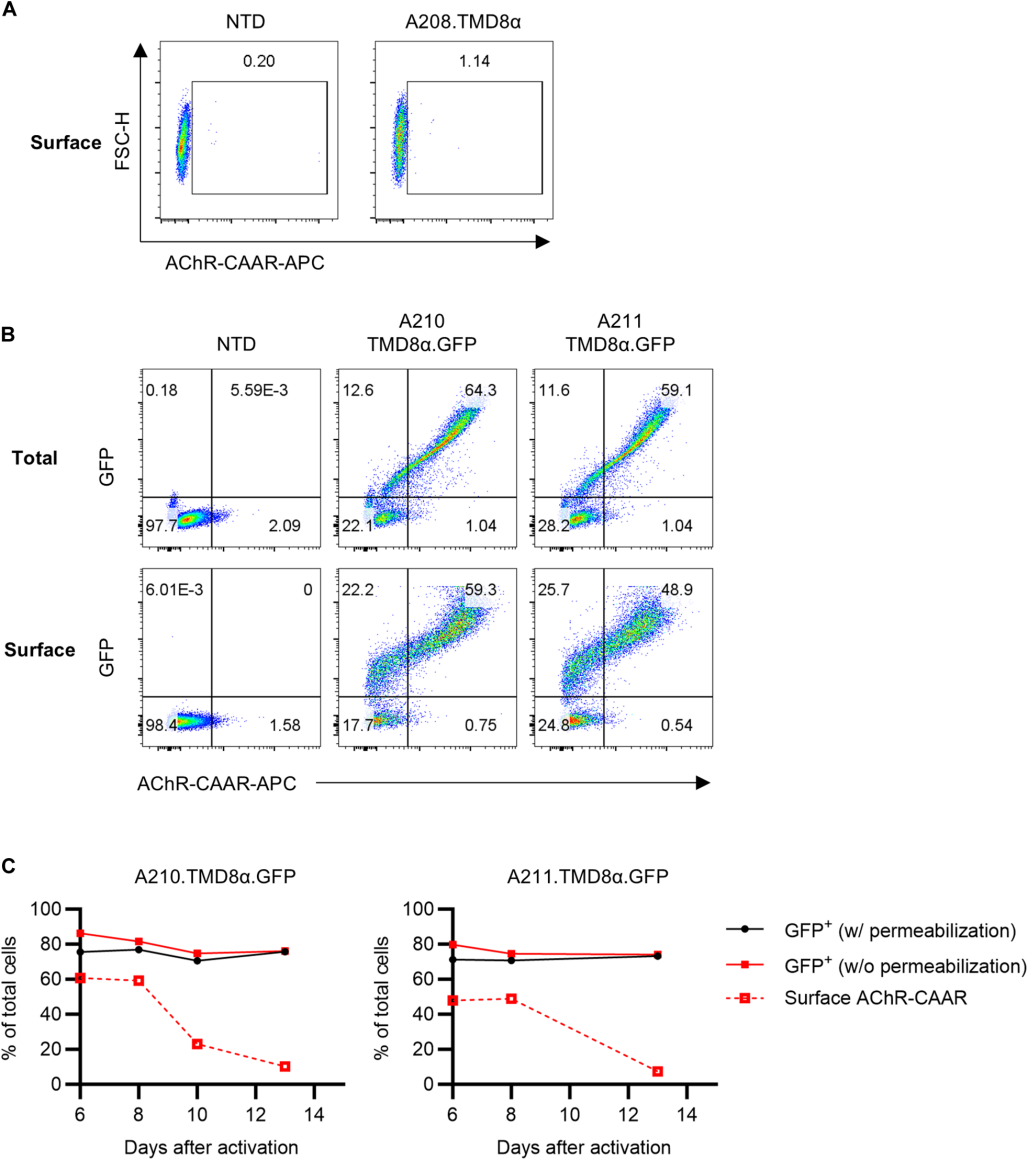
The expression pattern of A210.TMD8α-CAAR and A211.TMD8α-CAAR is comparable during ex vivo expansion. (**A**) Surface AChR-CAAR was stained using mAb 35 in A208.TMD8α-CAART. (**B** and **C**) Total and surface AChR-CAAR expression in A210.TMD8α.GFP-CAART and A211.TMD8α.GFP-CAART was detected with or without permeabilization before staining, respectively. Expression of AChR-CAAR and GFP is shown at day 8 after T cell activation in (B).

We first assessed the effects of membrane-proximal αEC1 residues on AChR-CAART surface expression. A short-form A208.TMD8α-CAAR (αEC1_1–208_) did not express on the surface of human T cells (Fig. 2A). To explore whether adding an extra amino acid residue in the TMD of the CAAR construct could improve the intracellular trafficking and surface expression stability, we compared CAAR expression between A210.TMD8α-CAAR and A211.TMD8α-CAAR (αEC1_1–211_) using a C-terminal green fluorescent protein (GFP) fusion. During ex vivo expansion, A211.TMD8α.GFP-CAAR demonstrated surface expression instability akin to that observed with A210.TMD8α.GFP-CAAR (Fig. 2, B and C).

**Fig. 3.**
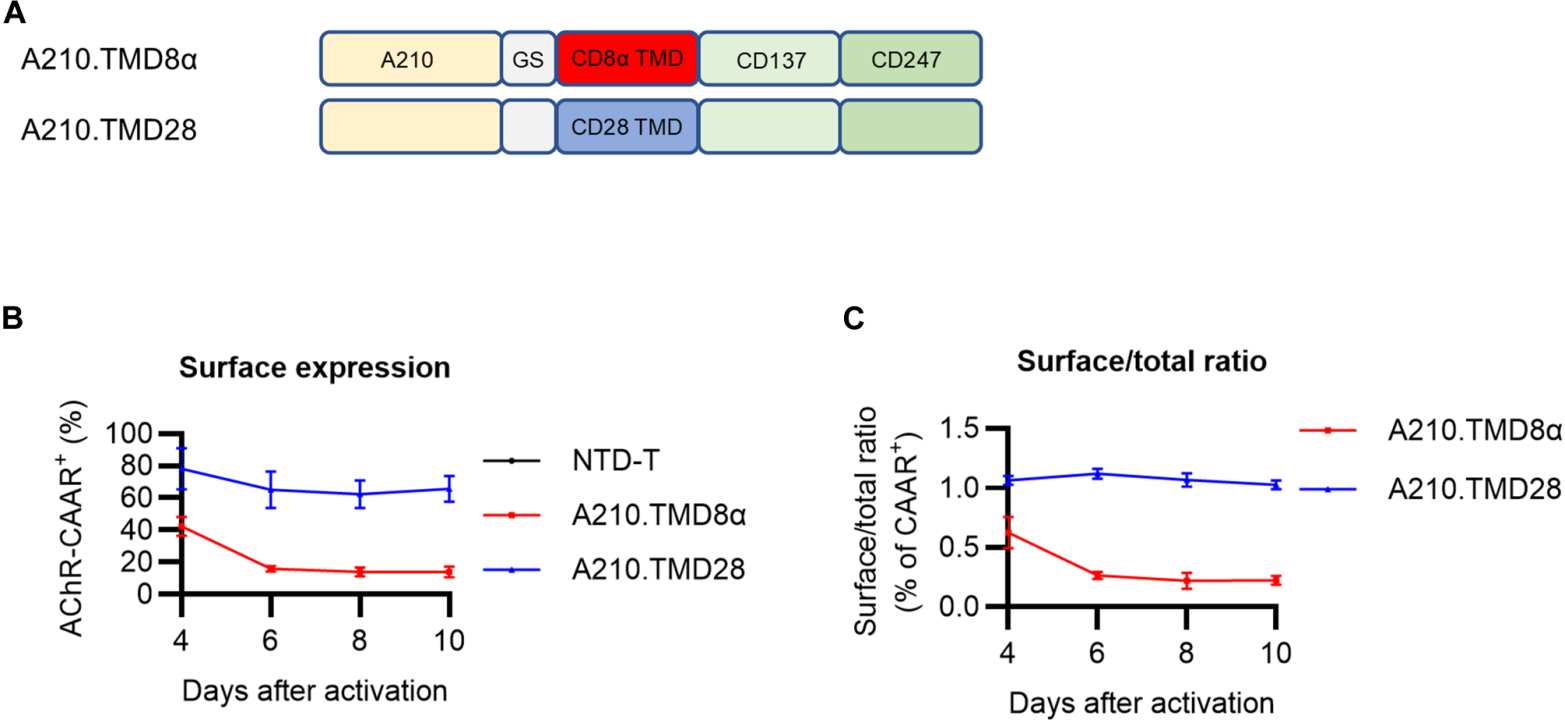
Incorporation of a CD28 TMD stabilizes AChR CAAR surface expression. (**A**) Schematic diagram sho0wing A210.TMD8α-CAAR T cells and A210.TMD28-CAAR T cells. (**B** and **C**) Total and surface AChR-CAAR expression from NTD T cells (black), A210.TMD8α-CAAR T cells (red), and A210.TMD28-CAAR T cells (blue) during ex vivo expansion was detected with or without permeabilization, respectively. (C) Ratio of surface to total AChR-CAAR expression. Each dot represents mean ± SD.

The AChR TMD has been shown to play a crucial role in the regulation of the intracellular trafficking of unassembled AChR α subunits (*21*). To test whether the CD8α TMD affects CAAR surface expression stability, we incorporated the CD28 TMD into the AChR-CAAR construct (Fig. 3A). Whereas the ratio of surface to total expression of A210.TMD8α-CAAR decreased over time, the A210.TMD28-CAAR exhibited sustained surface expression during ex vivo T cell expansion (Fig. 3, B and C).

### Inhibiting lysosomal degradation partially restores surface A210.TMD8α-CAAR expression

**Fig. 4.**
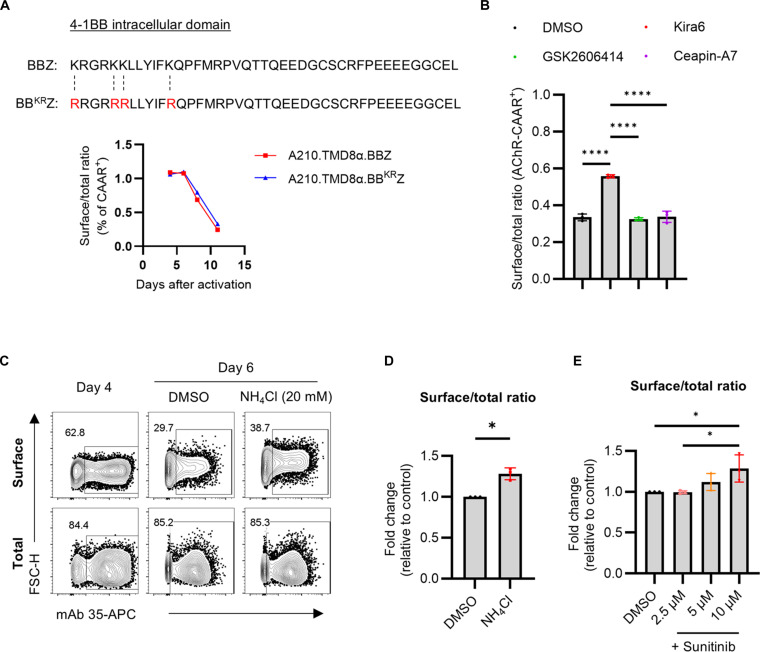
Inhibition of lysosomal degradation partially restores the surface expression of AChR-CAAR during ex vivo expansion. (**A**) The lysine residues within 4-1BB intracellular domains used in A210.TMD8α-CAAR were replaced with arginines (red). Surface AChR-CAAR expression relative to the total expression of A210.TMD8α.BBZ and A210.TMD8α.BB^KR^Z T cells was plotted. (**B**) A210.TMD8α-CAAR T cells were treated with each ER stress inhibitor (1 μM Kira6, 1 μM GSK2606414, or 6 μM Ceapin-A7) on day 4 and day 6. Total and surface AChR-CAAR expression was detected at day 7 with or without permeabilization before staining, respectively. Surface AChR-CAAR expression relative to the total expression was plotted. Each dot represents mean ± SD: ****P < 0.0001; one-way analysis of variance (ANOVA) with Holm-Sidak multiple comparisons test. (**C** and **D**) A210.TMD8α-CAAR T cells were treated with NH4Cl (20 mM) at day 4. Total and surface AChR-CAAR expression at day 6 is shown (C) and relative surface/total ratio of AChR-CAAR expression to nontreated A210.TMD8α-CAAR T cells was plotted (D). Each dot represents mean ± SD: *P < 0.05; paired *t* test. (**E**) A210.TMD8α-CAAR T cells were treated with sunitinib at indicated concentrations on day 4 and day 6. Surface and total AChR-CAAR expression was detected at day 7. Relative surface/total ratio of AChR-CAAR expression to the control (DMSO) was plotted. Each dot represents mean ± SD: *P < 0.05; one-way ANOVA with Holm-Sidak multiple comparisons test.

Activated CAR molecules undergo rapid ubiquitination and subsequent degradation by lysosomes (*22*). To evaluate whether disruption of ubiquitination-mediated protein degradation might also rescue the decline in surface A210.TMD8α-CAAR expression during ex vivo culture, we mutated intracellular lysine residues to arginines in the CD137 (4-1BB) and CD3ζ signaling domains (BBZ^KR^), which was previously reported to enhance recycling of internalized anti-CD19 CARs back to the cell surface (*22*). However, the A210.TMD8α.BBZ^KR^-CAAR mutant was not expressed on the surface of human T cells (fig. S4). Subsequently, we generated a mutant where only lysines in the 4-1BB intracellular domain were replaced with arginines (BB^KR^Z) (*23*). In contrast to BBZ^KR^, A210.TMD8α.BB^KR^Z-CAAR was successfully expressed on the surface, although its expression still downmodulated during ex vivo expansion (Fig. 4A). These data suggest that ubiquitination-mediated protein degradation may not be the primary cause of the unstable surface expression of A210.TMD8α-CAAR.

The AChR pentameric complex is assembled in the endoplasmic reticulum (ER), and unassembled AChR subunits are subject to degradation (*24*–*26*). In addition, ER stress is known to increase AChR endocytosis (*27*). Therefore, we investigated whether inhibitors of ER stress could rescue the decrease in CAAR surface expression levels. To test this, on day 7 after T cell activation, we treated A210.TMD8α-CAART with three different ER stress inhibitors: Kira6 [an inositol-requiring enzyme 1 alpha (IRE1α) inhibitor], GSK2606414 [a protein Kinase R-like endoplasmic reticulum kinase (PERK) inhibitor], or Ceapin-A7 [an activating transcription factor 6 (ATF6) inhibitor] (Fig. 4B) (*28*, *29*). The downmodulation of surface A210.TMD8α-CAAR expression was significantly mitigated by Kira6, whereas PERK and ATF6 inhibition had no effect on CAAR surface expression (Fig. 4B).

It is known that IRE1α senses misfolded protein and directs both proteasomal and lysosomal protein degradation (*30*). A210.TMD8α-CAART was treated with ammonium chloride (NH_4_Cl), an inhibitor of lysosomal function (*22*, *31*). Consistent with Kira6 effects, A210.TMD8α-CAART treated with NH_4_Cl showed a modest but statistically significant increase in surface versus total CAAR expression during ex vivo culture (Fig. 4, C and D). In addition, we tested sunitinib, which inhibits IRE1α kinase and lysosomal protein degradation (*32*). Similar to Kira6 and NH_4_Cl treatments, sunitinib attenuated the decrease in surface A210.TMD8α-CAAR in a dose-dependent fashion, although did not completely rescue CAAR surface expression (Fig. 4E).

### TMD dimerization motifs affect CAAR transport efficiency but not surface stability

CD8α molecules undergo dimerization at the ER before being transported to the plasma membrane, with this dimerization influenced by both the hinge domain and the TMD (*33*). CAR surface expression is also regulated by the hinge and the TMD (*34*). Considering the known role of cysteine residues in TMD structural stability (*35*), we investigated whether the cysteine residues in the CD8α TMD control the intracellular transport of A210.TMD8α-CAAR.

**Fig. 5.**
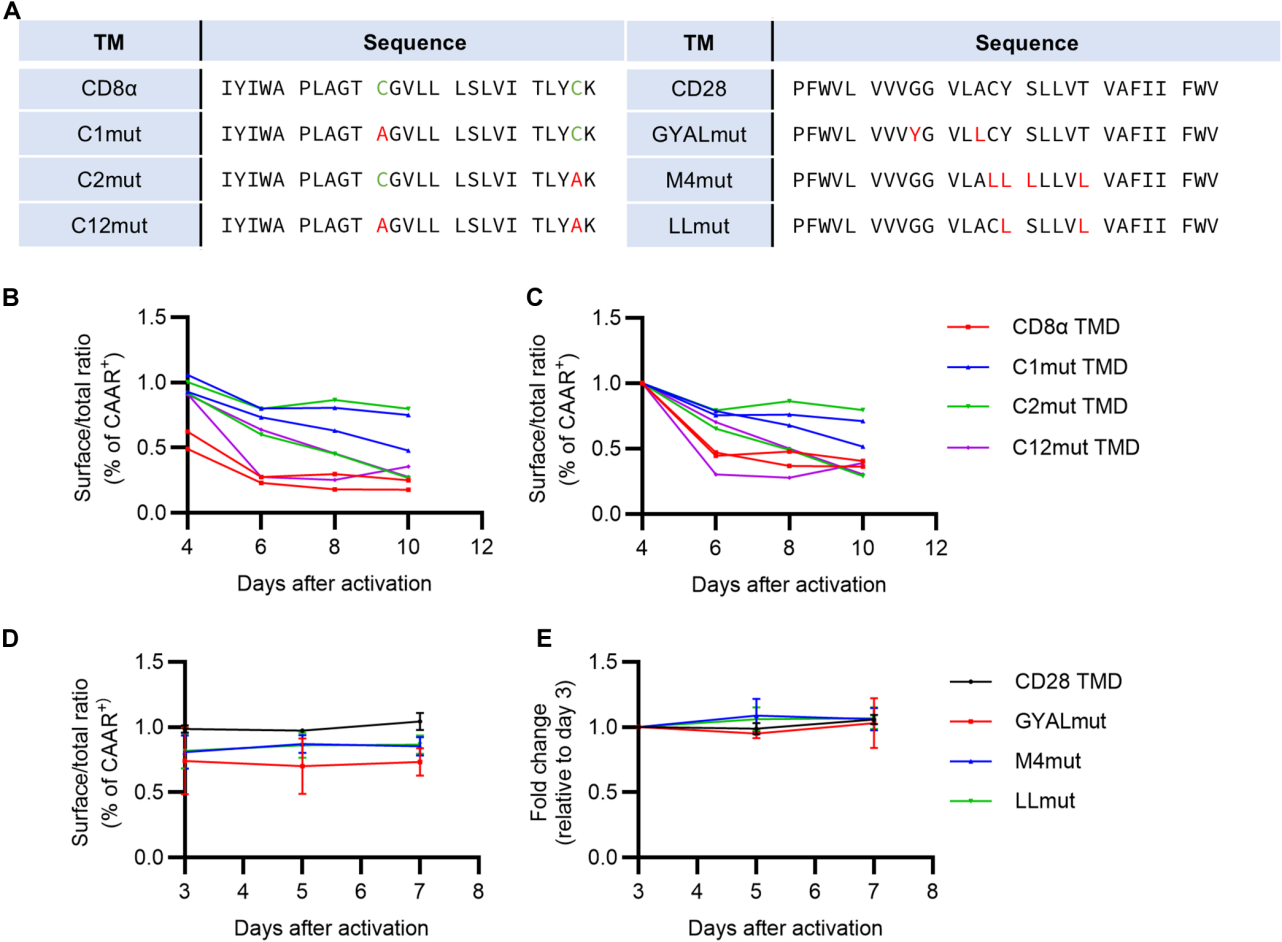
Dimerization motifs within TMDs affect the transport efficiency of A210-CAAR but do not influence the stability of surface expression. (**A**) Mutations of amino acid residues to disrupt homo- or heterodimerization of CD8α TMD or CD28 TMD are indicated in red. (**B** and **C**) Total and surface A210-CAAR expression from A210.TMD8α-CAAR T cells (red), C1mut (blue), C2mut (green), and C12mut (purple) during ex vivo expansion were detected with or without permeabilization before cell staining, respectively. Surface A210-CAAR expression relative to the total expression was plotted (B). Relative surface/total ratio of A210-CAAR expression to day 4 was plotted (C). This experiment was conducted using T cells from two different donors. (**D** and **E**) Total and surface A210-CAAR expression from A210.TMD28-CAAR T cells (black), GYALmut (red), M4mut (blue), and LLmut (green) during ex vivo expansion. Surface A210-CAAR expression relative to the total expression was plotted (D). Relative surface/total ratio of A210-CAAR expression to day 3 was plotted (E). Each dot represents mean ± SD.

There are two cysteine residues within the CD8α TMD (Fig. 5A). We replaced these two cysteine residues sequentially with alanines and evaluated CAAR surface expression. The efficiency of intracellular transport of AChR-CAAR molecules to the plasma membrane was enhanced in all cysteine mutants (C1mut, C2mut, and C12mut TMDs) (Fig. 5B). However, the fold change in the surface to total CAAR ratio was similar among all AChR-CAAR constructs (Fig. 5C). This suggests that cysteine residues in the CD8α TMD affect the intracellular transport efficiency of A210.TMD8α-CAAR but do not affect the stability of surface expression.

Subsequently, we investigated the importance of dimerization mediated by the CD28 TMD for A210.TMD28-CAAR surface expression. Three CD28 TMD mutants (Fig. 5A) known to prevent dimerization (*36*–*38*) were tested, and the surface to total CAAR expression ratio of these mutants was assessed. Consistent with prior findings (*36*, *38*), the surface expression of CD28 TMD mutants decreased CAAR surface expression levels (Fig. 5D). However, the surface expression of A210.TMD28-CAAR mutants was still sustained during ex vivo expansion (Fig. 5E), indicating that the mechanism of CD28 TMD rescue of CAAR expression does not depend on CD28 TMD-mediated dimerization.

### A210.TMD28-CAART exhibits comparable cellular cytotoxicity as A210.TMD8α-CAART

**Fig. 6.**
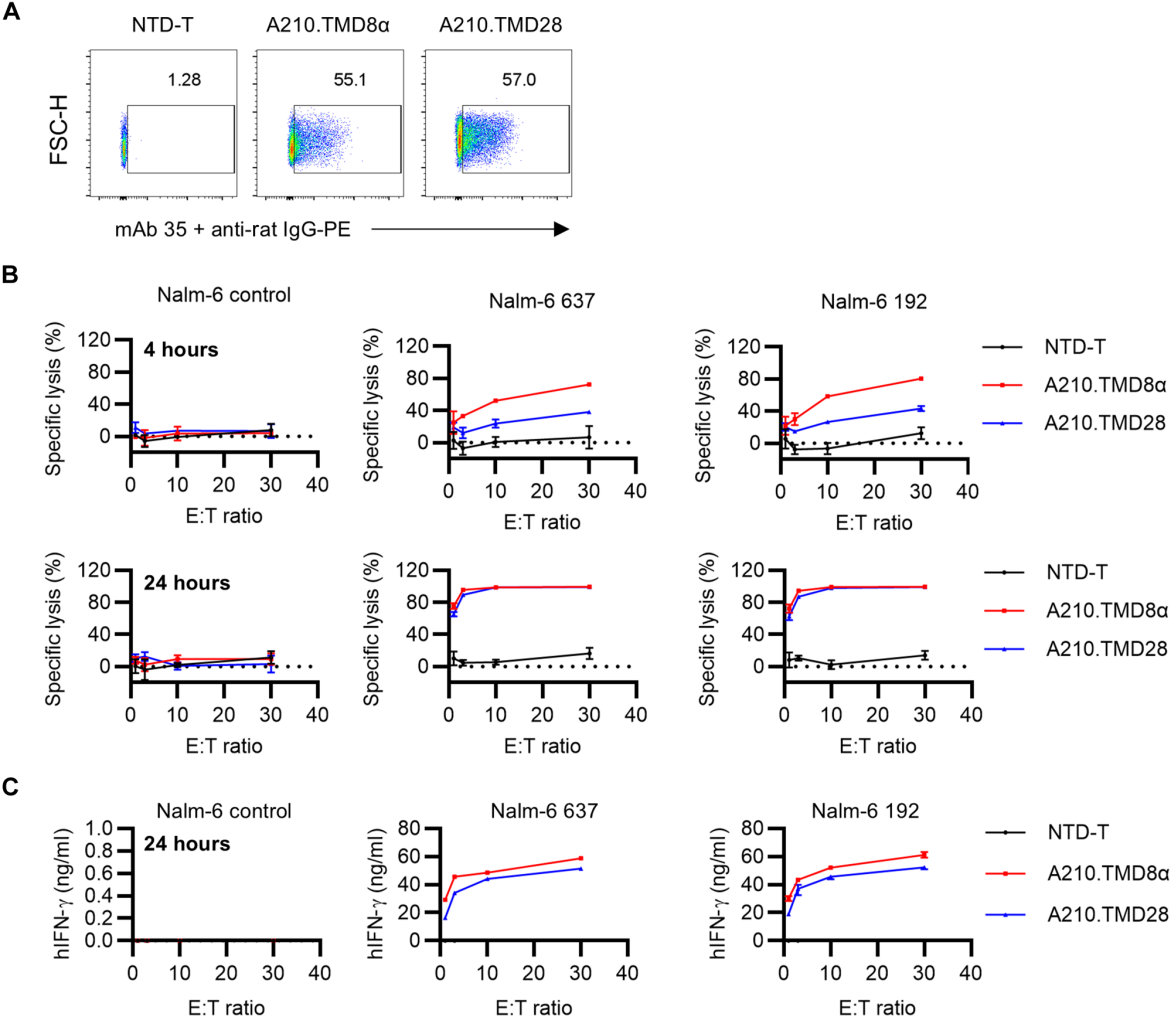
A210.TMD28-CAART exhibited slower but comparable cellular cytotoxicity with A210.TMD8α-CAART. (**A**) The percentage of AChR-CAAR^+^ cells of A210.TMD8α-CAAR T cells were detected using mAb 35 followed by staining by anti-rat IgG-PE. (**B**) AChR-CAAR T cells were coincubated with Nalm-6 control, Nalm-6 192, or Nalm-6 637 cells at indicated effector:target (E:T) ratios. Specific lysis (%) was detected at 4 hours and 24 hours after coincubation using a luciferase-based killing assay. (**C**) Supernatants were collected at 24 hours after coincubation, and human IFN-γ (hIFN-γ) production was detected using enzyme-linked immunosorbent assay. Each dot represents mean ± SD.

Comparing cytotoxicity between A210.TMD8α-CAART and A210.TMD28-CAART poses challenges due to their differing surface CAAR expression levels. We therefore sorted CAAR-positive cells in A210.TMD8α-CAART and adjusted the CAAR-positive cell ratio to be similar to A210.TMD28-CAART by diluting with donor-matched nontransduced (NTD) T cells (Fig. 6A). The cytotoxicity of A210.TMD8α-CAART and A210.TMD28-CAART against high-affinity (clone 637) and low-affinity (clone 192) anti-MIR Nalm-6 cells was then compared. There was no difference in cytotoxicity at 24 hours between A210.TMD8α-CAART and A210.TMD28-CAART, although the kinetics of cytolysis of target cells by A210.TMD28-CAART was slower than that of A210.TMD8α-CAART, assessed at 4 hours after target cell coculture (Fig. 6B). A210.TMD28-CAART exhibited comparable to slightly less interferon-γ (IFN-γ) production compared to A210.TMD8α-CAART at 24 hours (Fig. 5C). No cytotoxicity or IFN-γ production was detected against nontarget Nalm-6 cells for either construct (Fig. 6, B and C).

### A210.TMD28-CAART effectively controls target cell outgrowth in a mouse xenograft model

**Fig. 7.**
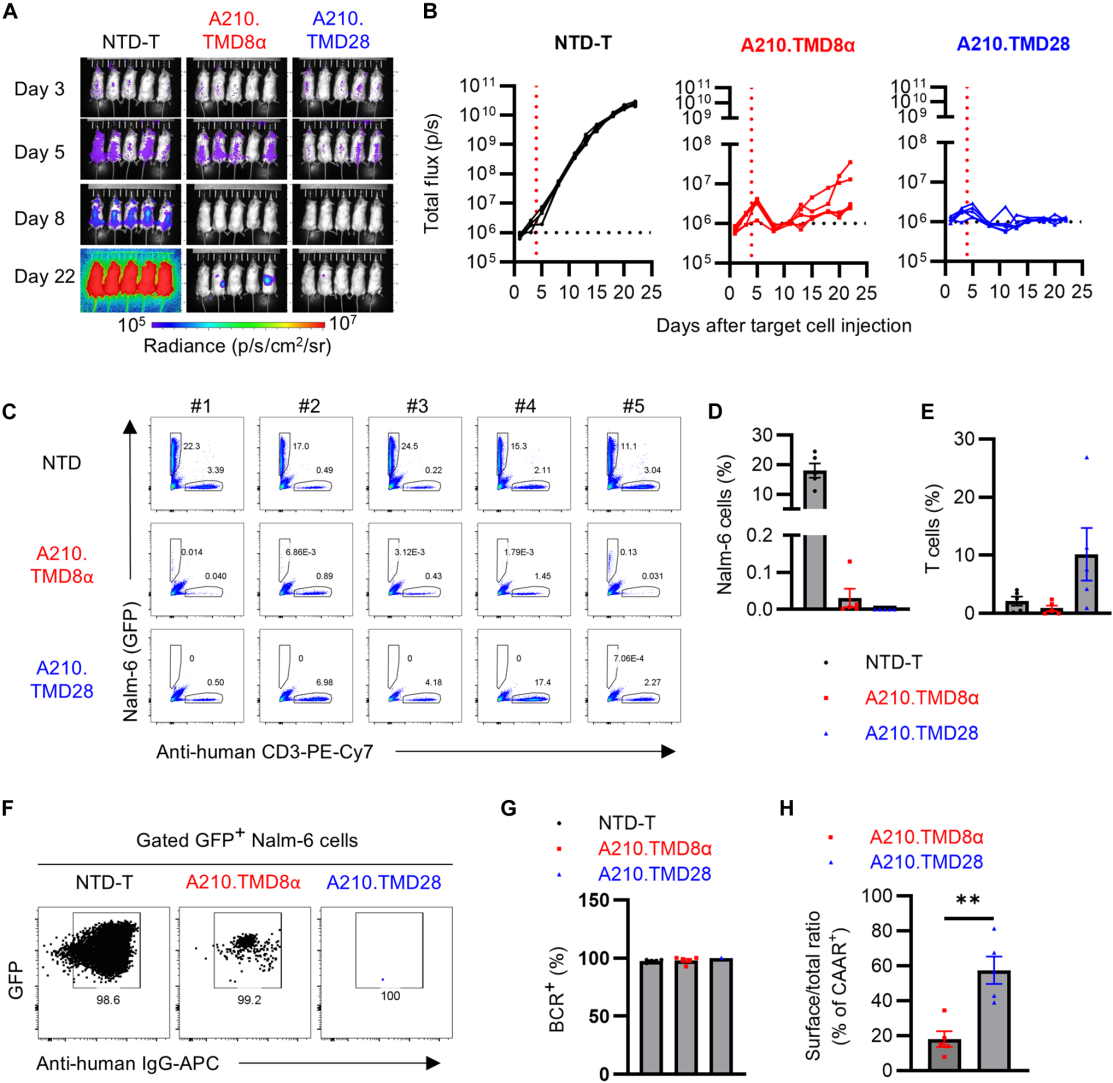
A210.TMD28-CAART demonstrates sustained cell surface expression and cytotoxic activity in vivo. (**A** and **B**) A total of 1 × 10^6^ Nalm-6 cells (1:1 mixture of Nalm-6 192 and Nalm-6 637 cells) were injected via tail vein at day 0. Mice were treated with 1 × 10^7^ T cells (*n* = 5, NTD-T; *n* = 5, A210.TMD8-CAART; and *n* = 5, A210.TMD28-CAART) on day 4 (indicated as a red dashed line) after target cell injection. Bioluminescence images were taken every 2 to 3 days, and total flux [photons per second (p/s)] was plotted. Basal level of total flux (1 × 10^6^) is indicated as a black dashed line. (**C** to **H**) Mice were euthanized after the final bioluminescence imaging. The profiles of Nalm-6 cells (GFP) and T cells (C) and IgG expression in GFP^+^ Nalm-6 cells (F) were plotted. The percentage of Nalm-6 cells (D), T cells (E), and IgG^+^ cells in Nalm-6 cells (G) and the surface/total ratio of AChR-CAAR expression in T cells (H) were summarized. Error bars show mean ± SEM: **P < 0.01; one-way ANOVA with Holm-Sidak multiple comparisons test.

In our in vitro data, A210.TMD8α-CAART lysed target cells more rapidly than A210.TMD28-CAART when they expressed the same level of surface CAAR expression (Fig. 6B). To directly compare their in vivo cytolytic activity, we adjusted CAAR-positive cells as described in Fig. 6A. Four days after injecting a mixture of Nalm-6 192 and Nalm-6 637 cells into NOD.scid.gamma (NSG) mice, mice were treated with either A210.TMD8α-CAART or A210.TMD28-CAART (Fig. 7, A and B; treatment day indicated by red dashed line). In the acute phase after infusion (between day 4 and day 8), both CAARTs effectively controlled target cell outgrowth (Fig. 7, A and B). However, Nalm-6 cells started to rebound between day 8 and day 13 after target cell injection in the A210.TMD8α-CAART–treated group, whereas no rebound was observed in the A210.TMD28-CAART–treated group (Fig. 7, A and B).

We examined the profile of Nalm-6 cells and T cells in the spleen of treated mice. Consistent with the bioluminescence imaging results (Fig. 7, A and B), the frequency of Nalm-6 cells in both CAART-treated mice was significantly lower than in NTD-T–treated mice (Fig. 7, C and D), with a small number of residual Nalm-6 cells detectable in five of five A210.TMD8α-CAART–treated mice versus one residual Nalm-6 cell detectable in one of five A210.TMD28-CAART–treated mice (Fig. 7, C and D). To ascertain whether the outgrowing Nalm-6 cells in CAART-treated mice remained targetable, we examined surface BCR expression in Nalm-6 cells. In contrast to our previous report demonstrating the escape of Nalm-6 cells by decreasing surface anti-MuSK BCRs in MuSK-CAART–treated mice (*12*), the majority of Nalm-6 cells found in CAART-treated mice expressed a comparable level of surface BCR expression relative to NTD-T–treated mice (Fig. 7, F and G). This suggests that antigen loss in Nalm-6 cells is not the reason for the rebounding bioluminescence flux.

Subsequently, we investigated whether T cells sustain CAAR surface expression. In this NSG mouse model, mice were treated with the same number of T cells exhibiting comparable surface CAAR positivity (55.1% in A210.TMD8α-CAART and 57.0% in A210.TMD28-CAART; Fig. 6A). The frequency of total T cells in A210.TMD8α-CAART–treated mice was lower than that of A210.TMD28-CAART–treated mice (Fig. 7E). In addition, the ratio of surface to total AChR-CAAR expression was significantly higher in A210.TMD28-CAART than A210.TMD8α-CAART (Fig. 7H). Collectively, these data indicate that the in vivo efficacy of AChR-CAAR T cells using the CD28 TMD is due to enhanced intracellular trafficking and stable surface CAAR expression.

## DISCUSSION

Previously, we reported the development of DSG3-CAART for mucosal pemphigus vulgaris and MuSK-CAART for MuSK-MG (*10*–*12*). Optimal CAAR design involves using an ectodomain that incorporates the key pathogenic epitopes of each autoantigen to deplete the full scope of autoreactive B cells that can cause disease. For MuSK-CAAR, we incorporated the entire extracellular domain sequence, covering from the first Ig-like domain to the Frizzled-like domain. DSG3-CAAR used a partial extracellular domain from EC1 to EC4, excluding EC5 where only nonpathogenic autoantibodies bind (*39*, *40*). Here, we have developed an AChR-CAART to target anti-αEC1 B cells. Adsorption studies using individual AChR subunits or antibody inhibition studies have shown that the majority of MG patient sera react against the α subunit compared to other subunits within the pentameric structure (*16*, *17*, *19*, *41*, *42*), underlying the rationale for targeting αEC1. One study found that 78, 20, and 36% of 86 AChR-MG patient sera were inhibited by 50% or more by anti-α, anti-β, and anti-γ antibodies, respectively (*41*), suggesting that the majority of autoantibodies target the α subunit. Another study found that the α subunit adsorbed up to 94% of AChR-reactive serum autoantibodies, with a mean of 35% adsorption across all 50 AChR-MG patient sera (*42*). Although other AChR subunits can be targeted, recent studies suggest that α-reactive autoantibodies are the most efficient at inducing MG pathology due to their polyfunctionality in causing receptor internalization, complement fixation, and/or inhibition of acetylcholine binding (*43*). In addition, autoantibodies targeting the α subunit have been proposed to cluster the AChR into linear arrays due to the presence of two α subunits (*44*); because effective complement fixation requires dense clusters of autoantibody binding on the cell surface (*45*), elimination of αEC1-reactive autoantibodies may be sufficient to eliminate such clusters and hence decrease neuromuscular junction complement fixation below the pathogenic threshold.

Engineering AChR-CAART poses a technical challenge because individual AChR subunits are not naturally expressed on the cell surface, owing to intrinsic ER retention signals. These subunits must first assemble to be exported to the plasma membrane, as the pentameric structure carries ER retention signals like the PLYFxxN motif in the first endogenous TMD, conserved across all AChR subunits (*21*). The role of the TMD in regulating intracellular trafficking appears crucial, as replacing the endogenous TMD with the epidermal growth factor receptor TMD restores surface expression of monomeric AChR α subunits (*21*). Our data indicate that αEC1 (A210) combined with a (GGGGS)_2_ linker and the CD8α TMD was successfully expressed on the human T cell surface and selectively eliminated anti-EC1α target cells, although CAAR surface expression gradually decreased during ex vivo expansion (Fig. 1A). CAR molecules can be degraded by either proteasomes or lysosomes (*46*–*48*). In the case of A210.TMD8α-CAAR, our data suggest that lysosomal degradation may play more of a role in inhibition of CAAR surface expression because inhibiting ubiquitination at the signaling domains does not restore CAAR surface expression, and lysosomal protein degradation inhibitors including ammonium chloride and sunitinib partially rescue surface A210.TMD8α-CAAR expression.

Unlike A210.TMD8α-CAAR, surface expression of A210.TMD28-CAAR is sustained during ex vivo expansion. Mutation of the dimerization motif within the CD28 TMD did not affect the stability of surface A210.TMD28-CAAR expression, although the efficiency of intracellular trafficking to the plasma membrane was slightly decreased. To our knowledge, no prior reports have shown that the TMD directly influences the stability of CAR surface expression. Our studies identify novel biological factors that affect the success of CAAR engineering and highlight the importance of longitudinal monitoring for stable CAAR surface expression. Full preclinical assessment of AChR-CAART would involve additional studies on pharmacology and potential off-target toxicities. The studies presented here lay the groundwork for future preclinical development of CAART technology for therapeutic applications in AChR-MG.

## MATERIALS AND METHODS

### Ethics statement for animal research

All studies involving animals were performed under a protocol approved by the University of Pennsylvania Institutional Animal Care and Use Committee (protocol #805652).

### Design and construction of plasmids

Cloning methods for lentiviral plasmids followed previously described methods (*12*). Briefly, the human CHRNA1 ectodomain (amino acids 21 to 230, UniProt P02708-2) was synthesized (Integrated DNA Technology) with flanking 5′ Bam HI and 3′ Nhe I restriction sites. Digested gene fragments were ligated into the pTRPE-MuSK-CAAR vector upstream of a spacer domain (GGGGS)_2_, the CD8α TMD, the CD137 costimulatory domain, and the CD3ζ signaling domain. For some constructs, other TMD gene fragments were synthesized with flanking 5′ Nhe I and 3′ Sal I restriction sites and then digested/ligated into pTRPE-A210-CAAR.TMD8α. The GFP sequences were fused to the C terminus of the CD3ζ signaling domain using a (GGGGS)_2_ linker to generate the construct A210.TMD8α.GFP and A211.TMD8α.GFP.

Anti-AChRα BCR 192 (rat) and 637 (human) were produced by synthesizing each variable heavy (VH) and variable light (VL) chain sequence with 5′ Bam HI/3′ Nhe I restriction sites for VH, 5′ Xho I/3′ Bsi WI (for 192 VL), and 5′ Xho I/3′ Bsu 36I (for 637 VL). Gene fragments were digested and ligated into the pTRPE-IgG4 (membrane-bound form) vector, linked via a P2A sequence to either Igκ or Igλ. The IGHV and IGLV sequences for 637 were obtained from GenBank X98215.1 and X98219.1, respectively (*19*). Before designing the synthetic gene fragments, the 5′ end of the variable regions was modified to include the entire FR1 regions, each of which encoded nucleotides for the best matched International ImMunoGeneTics Information System (IMGT)-listed germline genes, IGHV3-11*03 F and IGLV6-57*01 F (table S1).

### Lentivirus production

Packaging plasmids gag/pol/env (Nature Technology Corp or Aldevron) were used with Lipofectamine 2000 (Life Technologies) for lentiviral preparation in 293K cells or Lenti-X 293T cells (Takara, catalog no. 632180). Culture supernatants were collected at day 1 and day 2 after transfection, and viral particles were precipitated using high-speed centrifugation, reconstituted in culture media, and stored at −80°C for further experiments.

### Viral transduction and ex vivo expansion of CAAR T cells

Primary human T cells (University of Pennsylvania Human ImmunologyCore) were cultured in CTS OpTmizer media (Invitrogen, A1048501) plus 5% human AB serum (Gemini Bio-Products, 100-512), recombinant human interleukin-2 (rhIL-2) (100 IU/ml; Proleukin), 10 mM Hepes, 1% penicillin/streptomycin, and 1% GlutaMAX. T cells were activated and selected with anti-CD3/CD28–coated paramagnetic beads (Thermo Fisher Scientific, catalog no. 40203D) at a 3:1 bead:cell ratio. Lentivirus was added at 20 to 28 hours after activation, and cells were expanded with media changes every 2 to 3 days and magnetic bead removal before cryopreservation. For some experiments, AChR-CAAR T cells were stained with mAb 35–allophycocyanin (APC) and sorted using anti-APC microbeads (Miltenyi Biotec, catalog no. 130-090-855) and Miltenyi LS columns (catalog no. 130-042-401) following the manufacturer’s protocol.

### Luciferase-based in vitro cytotoxicity assay

Nalm-6 target cells expressing click beetle green luciferase (1 × 10^4^ cells per well in flat-bottom 96-well plates) were cocultured with engineered T cells or donor-matched NTD T cells at an indicated effector:target ratio. Two to 3 hours after coculture, luciferase substrate (d-luciferin potassium salt, GoldBio) was added to each well, and emitted light was measured (BioTek, Synergy HTX) at indicated time points. Percent-specific lysis was calculated using the luciferase activity of 5% SDS-treated cells as maximum cell death and media alone as spontaneous cell death using the formula: Specific lysis (%) = 100 × [(experimental data − maximum death data)/(maximum death data − spontaneous death data)].

### Inhibitor treatments

Kira6 (2 mM) in dimethyl sulfoxide (DMSO), 10 mM GSK2606414 in DMSO, and 10 mM Ceapin-A7 in DMSO were gifted from T. Ridky (University of Pennsylvania). Sunitinib malate (Cayman Chemical, catalog no. 13159-1) was reconstituted in DMSO at a 5 mM concentration. Ammonium chloride (NH_4_Cl) (Sigma-Aldrich, catalog no. 213330) was dissolved in ultrapure water at a 1 M concentration. Drugs were added every 2 days to fresh T cell culture media supplemented with 100 IU of rhIL-2.

### Flow cytometry detection and analysis

#### Detection of AChR-CAAR expression

mAb 35 (BioXCell, catalog no. BE0123) was conjugated with APC (Abcam, catalog no. ab201807) following the manufacturer’s protocol. To detect surface A210-CAAR expression, 5 × 10^6^/ml T cells were stained for 30 min on ice with mAb 35 or mAb 210 (2 μg/ml; Santa Cruz, catalog no. sc-58603), washed twice, and resuspended in phosphate-buffered saline (PBS). To detect total A210-CAAR expression, cells were fixed using eBioscience Fixation and Permeabilization Buffer (Thermo Fisher Scientific, catalog no. 88-8824-00) and stained with mAb 35–APC antibody for 30 min on ice. Cells were washed twice with permeabilization buffer and suspended in PBS for flow cytometer analysis.

#### Detection of Nalm-6 cells and human T cells in an NSG mouse xenograft model

Splenocytes were isolated by homogenizing the spleen between the frosted ends of the slides and then transferred to a 70-μm cell strainer (Falcon, catalog no. 352350). Cells were washed with PBS, resuspended in red blood cell (RBC) lysis buffer (BioLegend, catalog no. 420301), and then stained for 30 minutes on ice with phycoerythrin (PE)–Cy7 anti-human CD3 (clone UCHT1, Tonbo Bioscience, catalog no. 60-0038-T100), mAb 35–APC, and/or PE anti-human IgG (BD Biosciences, catalog no. 555787).

### Nalm-6 xenograft mouse model

#### Target cell and T cell injection

NSG mice (NOD.Cg-PrkdcscidIL2rgtm1Wjl/ SzJ) received intravenous Ig (600 mg/kg; Privigen, IVIg) via tail vein injection on day −2 and day −1 before target cell injection and every 2 to 3 days after T cell injection. On day 0, mice each received 1 × 10^6^ Nalm-6 target cell lines (1:1 mixture of Nalm-6 192 and Nalm-6 637) via tail vein injection. Donor-matched frozen human T cells (NTD-T, A210.TMD8α-CAART, and A210.TMD28-CAART) were thawed and incubated overnight in human T cell culture media supplemented with 100 IU of rhIL-2 before injection on day 4 after target cell injection (1 × 10^7^ T cells via tail vein, five mice per group).

#### Bioluminescence imaging

Bioluminescence was measured with a Xenogen IVIS Lumina S3 (Caliper Life Sciences) on day 1 after target cell injection and every 2 to 3 days thereafter by injecting d-luciferin potassium salt (GoldBio) intraperitoneally at a dose of 150 mg/kg. Mice were anesthetized with 2% isoflurane, and luminescence was serially measured at 1-min intervals until signals start to decrease in an automatic exposure mode. Total flux in the peak image was quantified using Aura Imaging Software v4.0.8 (Spectral Instruments Imaging) by drawing rectangles from head to 50% of the tail length. Radiance unit of p/s/cm^2^/sr = number of photons per second per square centimeter that radiate into a solid angle of one steradian.
