# Supplementary material for: Composition and function of AChR chimeric autoantibody receptor T cells for antigen-specific B cell depletion in myasthenia gravis
Source: Sci Adv. 2025 Feb 28;11(9):eadt0795. doi: 10.1126/sciadv.adt0795 (PMC11870065; doi:10.1126/sciadv.adt0795)
Supplement: Supplementary file 1 — Figs. S1 to S4 Table S1 [file sciadv.adt0795_sm.pdf]

Science Advances
AAAS

Supplementary Materials for

Composition and function of AChR chimeric autoantibody receptor T cells for antigen-specific B cell depletion in myasthenia gravis

Sangwook Oh *et al.*

Corresponding author: Sangwook Oh, swoh@hallym.ac.kr; Aimee S. Payne, asp2261@cumc.columbia.edu

Sci. Adv. **11**, eadt0795 (2025)
DOI: 10.1126/sciadv.adt0795

This PDF file includes:

Figs. S1 to S4
Table S1

Fig. S1.

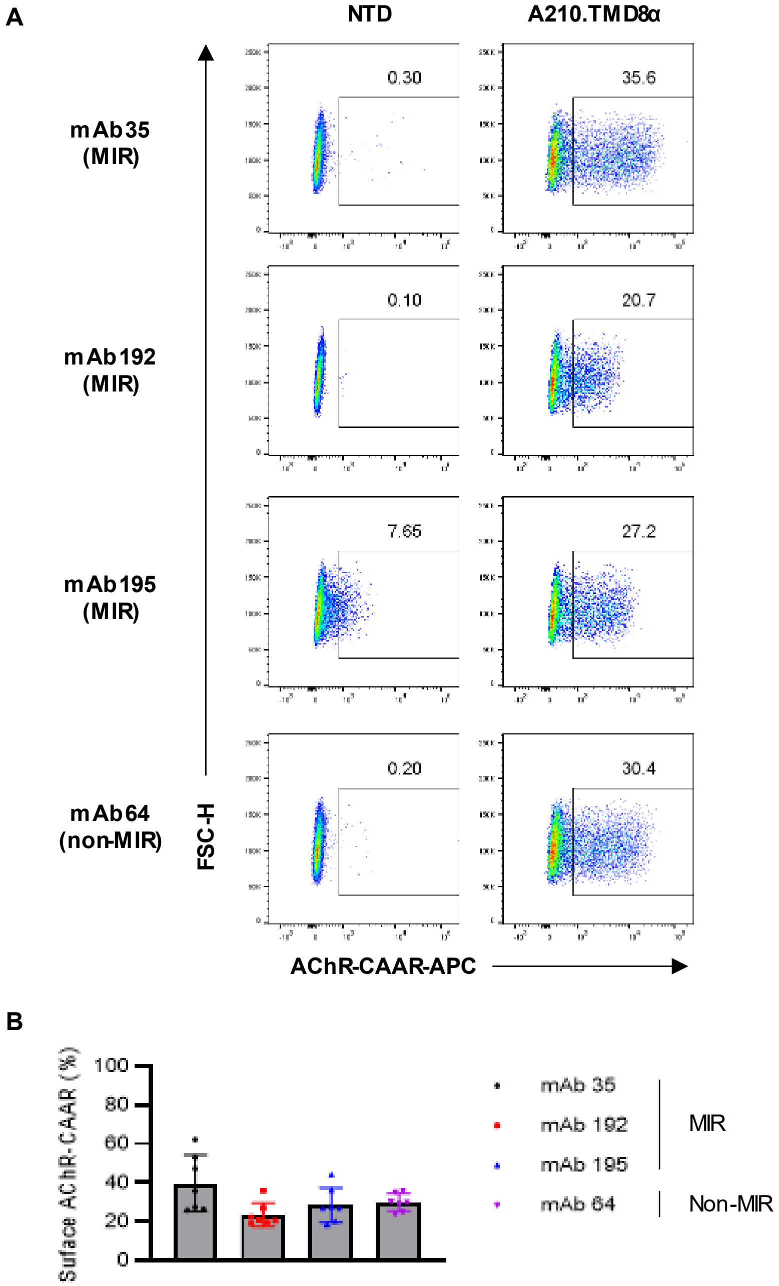

Detection of A210.TMD8α-CAAR expression using a panel of monoclonal antibodies targeting MIR or non-MIR residues.
(**A**) Flow cytometric analysis of A210.TMD8α-CAAR expression on the cell surface. Cells were stained with monoclonal antibodies specific to MIR (mAb 35, mAb 192, mAb 195) or non-MIR residues (mAb 64) of the αEC1 domain. The percentage of cells positive for surface AChR-CAAR binding is indicated in each plot. NTD, nontransduced. (**B**) Mean ± SD of surface AChR-CAAR expression (%) from independent experiments based on flow cytometric analysis.

Fig. S2.

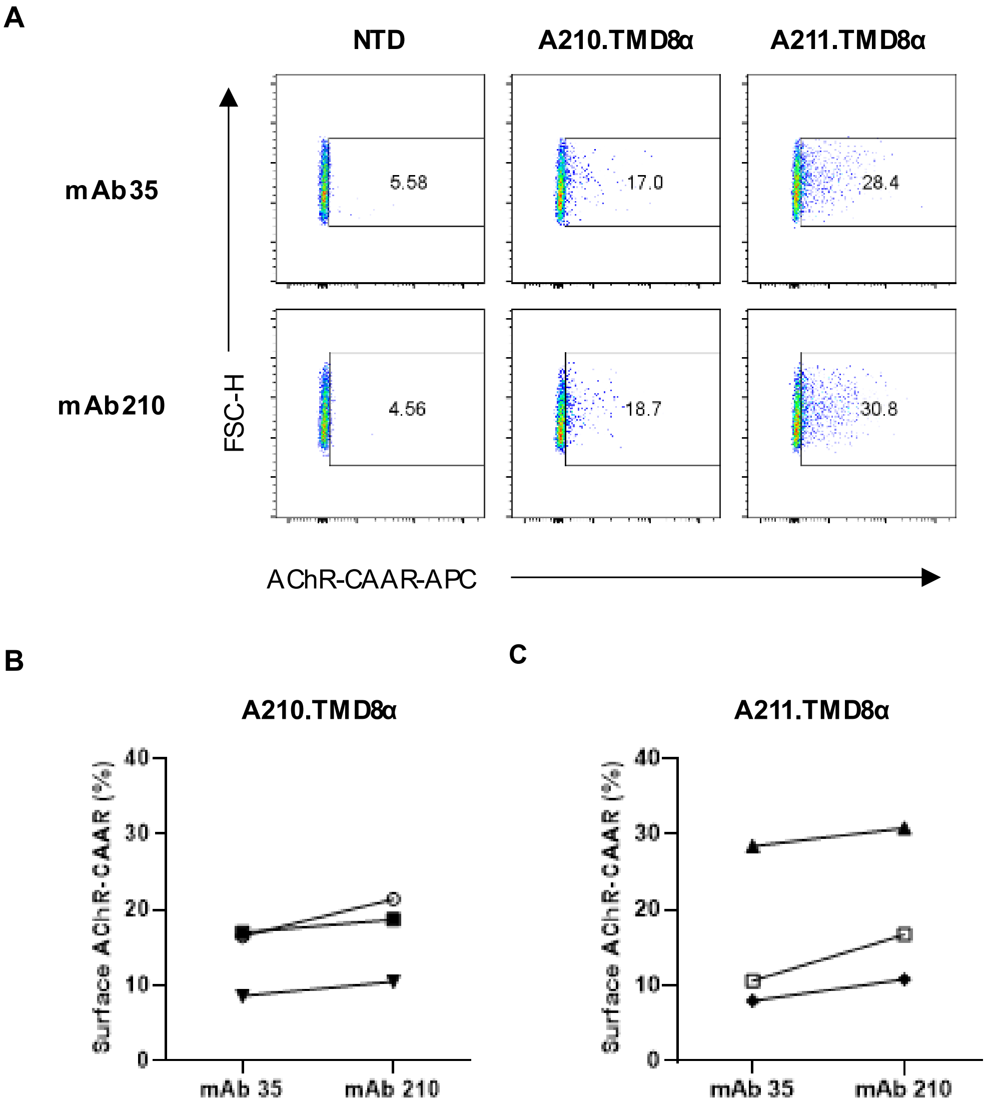

Analysis of A210.TMD8α-CAART and A211.TMD8α-CAART binding to conformation-dependent and conformation-independent anti-AChR antibodies.

(**A**) T cells expressing either A210.TMD8α-CAAR or A211.TMD8α-CAAR were stained with anti-AChR monoclonal antibodies that are either conformation-dependent (mAb 35) or conformation-independent (mAb 210). Binding was assessed using FACS to evaluate the differences in antibody recognition. (**B**, **C**) Summary of FACS data representing the percentage of AChR-CAAR positive cells from three different donors. Lines connect experiments conducted with cells derived from the same donor, indicating consistency across experimental conditions.

Fig. S3.

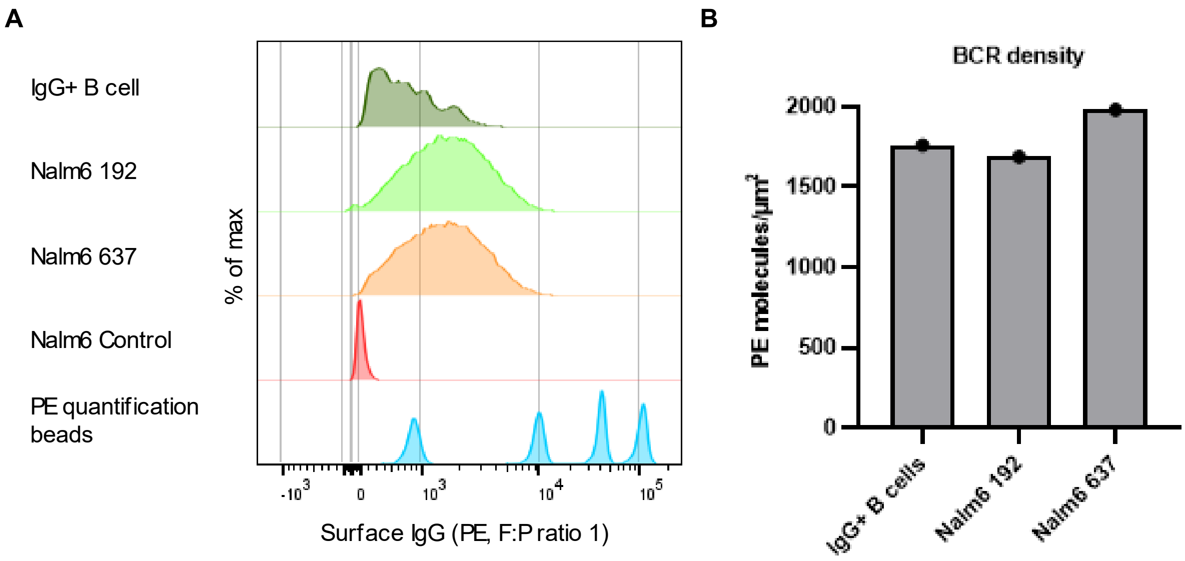

The BCR density of Nalm-6 637 and Nalm-6 192 is comparable to primary human IgG+ B cells.

(**A**) BCRs in primary human IgG^{+} B-cells and Nalm-6 cells expressing 637 or 192 BCRs were stained with PE mouse anti-human IgG. (**B**) BCR density was calculated by dividing the mean number of PE molecules/cell by the mean surface area (μm^2).

Fig. S4.

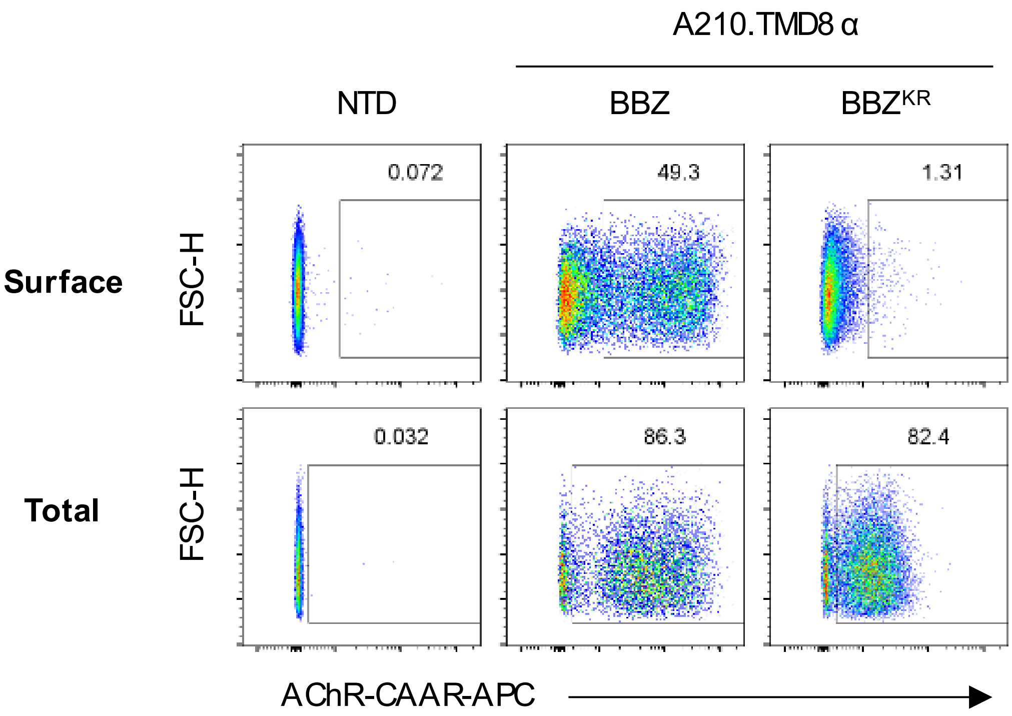

Mutation of cytoplasmic lysine residues inhibits A210.TMD8α-CAAR surface expression. Lysine residues within 4-1BB and CD3ζ cytoplasmic domains (BBZ) of A210 CAAR with a CD8α TMD were replaced with arginine (BBZ^{KR}). Total and surface AChR-CAAR expression was detected with and without permeabilization prior to staining, respectively.

Table S1.

637	Amino acid sequence
**VH**	QVQLLESGGGLVKPGGSLRLSCAASGFKSTDYYMAWVRQAPGRGLEWV SFISGRVFTNYTASVRGRFTVFREDDNTSVYLQMSRLRVEDTAVYYCARL RGIFRGPLKPLEYYFDLWGRGTLVTVSS
**VL**	NFMLTQPHSVSESPGKTVTISCTRSSGSIASNYVQWYQQRPGSSPTTVIY EDNQRPSGVPDRFSGSIDSSSNSASLTISGLKTEDEADYYCQSYDSNSGG RVFGGGTKLTVL

637	Nucleotide sequence
**VH**	CAGGTGCAGCTGTTGGAGTCTGGGGGAGGCTTGGTCAAGCCTGGAGG GTCCCTGAGACTCTCCTGTGCAGCCTCTGGATTCAAATCCACTGACTAC TACATGGCCTGGGTCCGCCAGGCTCCAGGGAGGGGGCTGGAGTGGG TCTCATTCATTAGTGGTCGTGTTTTCACAAACTACACCGCCTCTGTGAG GGGCCGATTCACCGTCTTCAGAGAGGACGACAACACCTCGGTGTATCT TCAGATGAGCCGCCTGAGAGTCGAAGACACGGCCGTCTACTACTGTGC GAGACTGCGGGGAATTTTTCGAGGTCCCCTCAAACCCCTAGAGTACTA CTTCGATCTCTGGGGCCGTGGCACCCTGGTCACTGTCTCCTCA
**VL**	AATTTTATGCTGACTCAGCCCCACTCTGTGTCGGAGTCTCCGGGGAAG ACGGTAACCATCTCCTGCACCCGCAGCAGTGGCAGCATTGCCAGCAAC TATGTGCAGTGGTACCAGCAGCGCCCGGGCAGTTCCCCCACCACTGTG ATCTATGAGGATAACCAAAGACCCTCTGGGGTCCCTGATCGGTTCTCTG GCTCCATCGACAGCTCCTCCAACTCTGCCTCCCTCACCATCTCTGGAC TGAAGACTGAGGACGAGGCTGACTACTACTGTCAGTCTTATGATAGCAA CTCTGGGGGGAGGGTGTTCGGCGGAGGGACCAAGCTGACCGTCCTA

Sequences of AChR-specific autoantibody 637.
Nucleotide and amino acid sequences of the full heavy and light chain variable regions of AChR-specific autoantibody 637 BCR that were used to generate the Nalm-6 637 target cell line.
